# The accuracy of medical dispatch - a systematic review

**DOI:** 10.1186/s13049-018-0528-8

**Published:** 2018-11-09

**Authors:** K. Bohm, L. Kurland

**Affiliations:** 1 Department of Clinical Science and Education, Södersjukhuset, Karolinska Institutet, SE 118 83 Stockholm, Sweden; 2 Department of Emergency Medicine, Södersjukhuset, Stockholm, Sweden; 3 Department of Medical Sciences, Örebro University, Örebro, Sweden; 4 Department of Emergency Medicine, Örebro University Hospital, Örebro, Sweden

**Keywords:** Emergency medical dispatch, Emergency medical services, Medical order entry systems

## Abstract

**Background:**

It is a challenge to dispatch Emergency medical Services (EMS) appropriately with limited resources and maintaining patient safety; this requires accurate dispatching systems. The objective of the current systematic review was to examine the evidence, according to GRADE, for medical dispatching systems to accurately dispatch EMS according to level of acuity and in recognition of specific conditions.

A systematic search was performed trough PubMed, Web of Science, Embase (free text in all fields), Centre for Reviews and Dissemination (CRD), and Cochrane Central Register of Controlled Trials up to 16th of May, 2017. A combination of keywords and Medical Subject Heading (MeSH) terms relevant to “emergency medical dispatch criteria” were used, to search for articles published between 2012 and 2017. Publications were included according to the inclusion/exclusion criteria using the Systematic Reviews and Meta-Analyses (PRISMA) protocol. Level of evidence was evaluated in accordance with Grading of Recommendations Assessment, Development and Evaluation (GRADE). Articles included were those that provided evidence for at least one of the measures of dispatch system accuracy; i.e. sensitivity, specificity, positive and negative predictive and/or over- and under-triage. The search identified 1445 articles. After the removal of duplicates, 382 titles were reviewed for relevance and an additional 359 articles were excluded based on manuscript title and abstract. An additional five articles were excluded after review of the full text versions of the remaining articles. The current review included 18 publications which all were based on primary research.

**Conclusions:**

The 18 articles addressed the identification of cardiac arrest, stroke, medical priority and major trauma using different dispatching systems. The results of the current review show that there is a very low to low overall level of evidence for the accuracy of medical dispatching systems. We suggest that it is necessary to create a consensus on common standards for reporting before consensus can be reached for the level of accuracy in medical dispatching systems.

## Background

The objective for the telecommunicator at the dispatch center is - based on the information obtained during a telephone call – to evaluate whether emergency medical services (EMS) are needed and with which priority the resource needs to be dispatched [1]. The challenge is to dispatch EMS appropriately with limited resources and still be safe for the patients; this requires accurate dispatching systems.

There are several types of dispatching systems but they can be categorized as two types of systems; the Medical Priority Dispatch system (MPDS) [2, 3] mainly used in Anglo-Saxon countries, and the criteria-based dispatch (CBD) [4, 5] used in Nordic and European countries. Common for both systems is that the telecommunicator allocates each call to one of the listed chief complaints. While MPDS is based on codes and scripted questions to put to the caller, the CBD system relies on the experience of the telecommunicator to conduct the interview. In addition to the different systems for medical dispatching, there are also different systems for the EMS response. The EMS organization can have e.g. advanced and/or basic life support ambulances, first responders or pre-hospital emergency physicians and helicopter emergency services (HEMS). However, the accuracy of EMS systems, which per definition includes both dispatching and the response to dispatching are not systematically described.

Dispatching accuracy, or effectiveness, relates to the ability of the dispatching system to discriminate between the required EMS resources and the priority of these. Measures of accuracy are both discriminative, e.g. sensitivity and specificity, and predictive, e.g. positive predictive value and negative predictive value [6]. Other relevant measures of performance of dispatching systems are over- and under-triage [7]. While our systems are geared towards over-triage so as not to miss critical patients in need of medical interventions, i.e. to avoid under-triage, over-triage consumes resources and increases the risk for occupational injuries of health care personnel. There is, however, no consensus on levels for over-and under-triage or dispatching accuracy.

The objective of the current systematic review was to examine the evidence, according to GRADE, for medical dispatching systems to accurately dispatch EMS according to level of acuity and in recognition of specific conditions. Accuracy was measured as sensitivity, specificity, positive and negative predictive value in addition to over- and under-triage.

## Methods

### Search strategy

The current systematic review includes the identification of articles according to Preferred Reporting Items for Systematic Reviews and Meta-Analyses (PRISMA) criteria [8]. The identification of publications included in the current review was made through a systematic search of the PubMed, Web of Science, Embase (free text in all fields), Centre for Reviews and Dissemination (CRD), and Cochrane Central Register of Controlled Trials up to 16th of May, 2017. A combination of keywords and Medical Subject Heading (MeSH) terms relevant to “emergency medical dispatch criteria”, published in the last 5 years, was used with the assistance of a librarian (Table 1).

**Table 1:** Search string.

PubMed	
1	(medical[all fields] AND dispatch*[all fields]) OR (emergency[all fields] AND dispatch*[all fields]) OR “Emergency Medical Dispatch”[all fields] OR dispatch centres[all fields]
2	triage[all fields]
3	Criteria based[all fields]
4	Physician based[all fields]
5	“emergency medicine” [all fields]
6	2 OR 3 OR 4 OR 5
7	1 AND 6
8	English, year> = 2012
Web of Science (*Indexes = SCI-EXPANDED, SSCI, A&HCI, ESCI Timespan = All years)*	
1	Topic = (Emergency OR Medical OR centres) AND Dispatch*
2	Topic = triage
3	Topic = criteria based
4	Topic = physician based
5	Topic “emergency medicine”
6	2 OR 3 OR 4 OR 5
7	1 AND 6
8	7 AND English, year > = 2012, Article, Review
Embase (free text in all fields)	
1	(emergency OR medical OR centres) AND dispatch*
2	Triage
3	criteria based
4	physician based
5	“emergency medicine”
6	2 OR 3 OR 4 OR 5
7	1 AND 6
8	7 AND English, year > = 2012, article, review, article in press
CRD - Centre for Reviews and Dissemination, York	
1	Dispatch*, english, year > = 2012
Cochrane	
1	(emergency OR medical OR centres) AND dispatch*
2	Triage OR criteria OR physician OR “emergency medicine”
3	1 AND 2
4	3 AND english, year > = 2012, NOT conference

### Inclusion- and exclusion criteria

Publications were included in the systematic review if they presented primary data which evaluated the accuracyof medical dispatch systems in current use and provided evidence for at least one of the measures of dispatch system accuracy; i.e. sensitivity, specificity, positive predictive value (PPV) and negative predictive value (NPV) and/or over- and under-triage. Publications evaluating dispatch/ triage for military resources, mass casualty/disaster and inter-facility transfers were excluded. The search was limited to studies on humans, published in English.

### Title and abstract screening

The titles and abstracts were screened independently by the two authors. Inclusion in the subsequent full-text review was made through discussion and consensus.

### Full text screening for relevance

The selected full-text articles were reviewed independently on the basis of inclusion and exclusion criteria. Relevant articles were reviewed to determine whether they provided evidence for at least one of the measures of dispatch system accuracy; i.e. sensitivity, specificity, positive predictive value (PPV) and negative predictive value (NPV) and/or over- and under-triage.

### Level of evidence according to GRADE

Publications were reviewed in detail and the overall quality of evidence was based on the recommendations of the Grading of Recommendations Assessment, Development and Evaluation (GRADE) working group [9]. The level of evidence was categorized as ‘very low’, ‘low’, ‘moderate’, ‘high’ or ‘very high’ in accordance with GRADE [10] with special emphasis on diagnostic tests [10]. Briefly; retrospective studies are graded as very low or low, while high or very high require a prospective study design [10]. Factors that determine and can decrease the quality of evidence are study design, risk of bias, indirectness, inconsistency in study results, imprecise evidence and publication bias [10].

### Measurement of inter-rater agreement

The kappa coefficient was calculated to study the agreement between the observers ability to classify titles and abstracts (yes/no) [11].

## Results

### Study selection

**Fig. 1:**
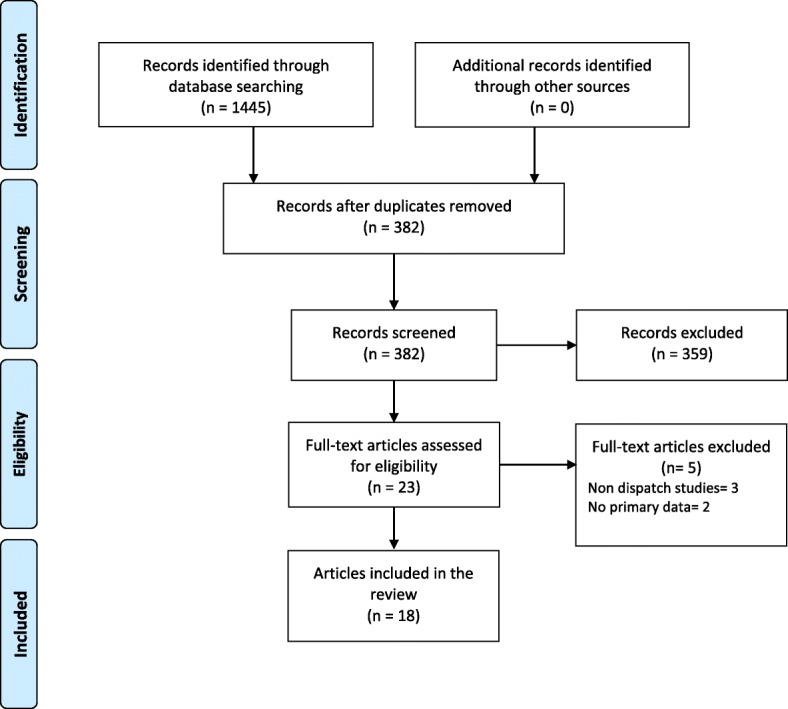
PRISMA flow diagram

The search identified 1445 publications. After the removal of duplicates, 382 titles were reviewed for relevance and an additional 359 citations were excluded based on manuscript title and abstract. An additional five articles were excluded (three non-dispatch studies and two with no primary data) after review of full text of the remaining publications. The current review included 18 publications. The PRISMA flow diagram summarizes the inclusion/exclusion process, Fig. 1.

### Inter-rater agreement

The k values, were 0.53 (95% CI; 0.45–0.62) for comparison of titles and 0.68 (95% CI; 0.50–0.86) for comparison of abstracts. The latter is considered as ‘substantial’ agreement between the raters [12].

### Characteristics of included articles

Characteristics of the 18 included publications are presented in Table 2. All included publications were primary research. It was not possible to perform a meta-analysis due to the heterogeneity of the studies included in the current systematic review. The results are presented below in relation to their study populations and main objectives, i.e. identification of cardiac arrest, stroke, medical priority and helicopter medical services dispatching for major trauma.

**Table 2:** Study characteristics included in systematic review

First author (year of publication)	Design	GRADE Rating	Area and/or Country of publication	Population	Number of patients/incidents	Dispatching system category	Additional information
Clawson J. J. et al. (2016) [25].	Retrospective descriptive	Very low	Salt Lake City, USA	EMD identified strokes	4712 hospital confirmed strokes	Medical Priority Dispatch System	Final inhospital diagnosis as stroke was reference
Dami F. et al. (2017) [22].	Retrospective observational	Very low	Region of Vaud, Switzerland	Identification of acute stroke, onset within 5 h	427 patients	Criteria Based Dispatch	Final inhospital diagnosis as stroke was reference
Malekzadeh J. et al. (2015) [24].	“Quasi empirical design”	Moderate	Mashhad, Iran	Suspected stroke among callers	246 patients	CPSS vs. “Regional system”	Final inhospital diagnosis as stroke was reference
Krebes S. et al. (2012) [26].	Retrospective observational	Low	Berlin, Germany	Emergency calls due to stroke	207 patients	MPDS with a new developed algorithm	Final inhospital diagnosis as stroke was reference
Viereck S. et al. (2016) [23].	Retrospective observational	Very low	EMS Copenhagen	Emergency calls due to stroke	2653 patients	Criteria Based Dispatch	Final inhospital diagnosis as stroke/TIA was reference
Deakin C. D. et al. (2017) [17].	Retrospective observational	Very low	United Kingdom	Emergency calls due to CA, adult	469,400/8830 emergency calls, adult	NHS Pathways	Using ambulance crew’s decision as reference
Moller T. P. et al. (2016) [18].	Retrospective observational	Very low	Denmark and Sweden	Patients from national cardiac arrest registers and connected emergency calls	776 patients from Denmark and 346 from Sweden	Criteria Based Dispatch	The information from the ambulance crew (cardiac arrest register) was reference
Fukushima H. et al. (2015) [19].	Before/after comparison	Very low	Japan	Patients from national cardiac arrest register and connected emergency calls	478 patients (before) and 427 (after)	“Regional system”	The information from the ambulance crew (cardiac arrest register) was reference
Tanaka Y. et al. (2014) [20].	Prospective observational	Very low	Japan	Emergency calls due to CA and connected ambulance records	2747 emergency calls with dispatcher-assisted cardiopulmonary resuscitation attempt	“Regional system”	The information from the fire department crew (cardiac arrest register) was reference
Vaillancourt C. et al. (2015) [16].	Retrospective observational	Very low	Canada	Emergency calls due to CA	2260/1536 emergency calls	DPCI	The information from the ambulance crew (cardiac arrest register) was reference
Gellerstedt M. et al. (2016) [21].	Retrospective observational	Low	Vätragötland, Sweden	Emergency calls due to chest pain	2285 consecutive patients dialed 112 with chest pain	Criteria Based Dispatch	Inhospital diagnosis as acute coronary syndrome was reference
Giannakopoulos G. F. et al. (2012) [15].	Retrospective	Very low	Netherlands	Trauma-related dispatch	420 trauma patients	Based on MOI	Identification of major trauma due to definitions
Wilmer I. et al. (2015) [27].	Retrospective observational	Low	London’s Air Ambulance	Major trauma	2203 helicopter activations	Closest to CBD, but there is no formal protocol	Identification of patients with serious injury due to definitions
Ball S. J. et al. (2016) [28].	Retrospective observational	Low	Australia, Perth Western Australia	Consecutive cases of ambulance dispatch	211,473 consecutive cases of ambualnce dispatch, “whole of population study”	Medical Priority Dispatch System	Time critical condition by paramedic-determined patient condition at the time of departing the scene was used as reference
Dami F. et al. (2015) [13].	Retrospective observational	Very low	Switzerland	Primary missions	29,008 ambulance missions	Criteria Based Dispatch	The severity of cases assessed by paramedics on site using the NACA-score was reference
Ek B. et al. (2013) [29].	Retrospective	Very low	Jämtland, Sweden	Consecutive cases of medical dispatch	4835 ambulance dispatches	Criteria Based Dispatch	METTS-A according to ambulance was reference
Leopardi M. and Sommacampagna M (2013) [30].	Retrospective observational	Very low	Italy	Emergency calls	53,606 emergency calls	“Regional system”	The sensitivity of subjective experience-based nurse dispatch in detecting the need for phycisian interventions
Moser A. et al. (2017) [14].	Before/after comparison	Very low	Switzerland	Emergency calls and connected ambulance records	27,886 (before) and 38,748(after)	Criteria Based Dispatch	Severity of cases assessed by paramedics on site using the NACA-score was used as reference

*CA* Cardiac arrest, *CPSS* Cincinnati Prehospital Stroke Score, *DPCI* Dispatch Priority Card Index, *HEMS* Helicopter Emergency Medical Service, *INT* Paramedic interrogation of caller, *MOI* Mechanism of injury, *NHS* National Health System, *REQ* Land ambulance request

The main results; the accuracy for dispatching systems is presented in Table 3, along with the results for over- and under-triage. Three articles presented all measures [13–15].

**Table 3:** Measures for dispatch accuracy per included study

Category	Author, year	Sensitivity % (95% CI)	Specificity % (95% CI)	PPV % (95% CI)	NPV % (95% CI)	Over-triage % (95% CI)	Under-triage % (95% CI)
Stroke	Clawson et al., 2016	86.4	26.6	20.0	90.2		
	Dami et al., 2017	67.8 (54.3–79.4)	98.6 (98.4–98.7)	9.4 (6.6–12.	99.9 (99.9–99.9)		
	Malekzadeh et al., 2015					11.6 vs 20.8	10.7 vs 13.6
	Krebes et al., 2012	Stroke: 53.3 (47.0–59.0)	97 (97–98)	Stroke: 47.8 (42.0–54.0)	98 (97–98)		
	Viereck et al., 2016	66.2 (64.4–68.0)		30.2 (29.1–31.4)			
Cardiac arrest	Deakin et al., 2017	75.9 (74.3–77.3)	98.6 (98.6–98.7)	26.8 (25.88–27.7)	99.8 (99.82–99.85)		
	Möller et al., 2016	Copenhagen 80.7 (77.7–84.3), Skåne 86.0 (81.3–89.8)					
	Fukushima et al., 2015	93	50				
	Tanaka et al., 2014	72.9 (71.7–74.1)	99.6 (99.6–99.6)				
	Vaillancourt et al., 2015	65.9 (63.5–68.2)	32.3 (29.0–35.9)	67.4	30.9		
Acute coronary syndrome	Gellerstedt et al., 2016	82.6					
Major trauma	Giannokopoulos et al., 2012	87.7	45.3	48.4	86.3	44	20.6
	Wilmer et al., 2015	MOI + INT: 80.2				MOI: 41.2	REQ/all: 19.7
						INT: 30.2	
						REQ: 27.7	
Medical priority	Ball et al., 2016	93.32 (92.71–93.89)	48.6 (48.45–48.89)				
	Dami et al., 2015	86 (85.6–86.4)	48 (47.4–48.6)	21.7 (21.2–22-2)	95.4 (95.2–95.6)	78	4.6
	Ek et al., 2013	93.32	15.4				
	Leopardi et al., 2013	78.0 (76.9–79.1)	83.8 (83.4–84.1)	36.6 (35.8–37.5)	96.9 (96.8–97.1)		
	Moser et al., 2017	A + B 86.8 (86.5–87.1)	A + B 67.4 (66.9–67.9)	29.2 (28.7–29.7)	97.0 (70.3–71.3)	After 70.8 (70.3–71.3)	After 3.0 (2.8–3.2)

*CI* Confidence Interval, *DPCI* Dispatch Priority Card Index, *HEMS* Helicopter Emergency Medical Service, *INT* Paramedic interrogation of caller, *MPDS* Medical Priority Dispatch System, *MOI* Mechanism of injury, *NHS* National Health System, *NPV* Negative Predicted Value, *PPV* Positive Predicted Value, *REQ* Land ambulance request

### Identification of cardiac arrest

The overall sensitivity for identifying cardiac arrest was 65.9% [16], 75.9% [17], 80.7 and 86.0% at two different sites [18], respectively. In two studies, the sensitivity was 93.0% [19] and 72.9% [20] after implementation of modified protocols. These five organizations used five different systems/protocols; NHS Pathways [Deakin], Criteria Based Dispatch (CBD) [20] and Dispatch Priority Card Index (DPCI) [16], and two different Japanese protocols [19, 20]. The corresponding specificity was 32.3% [16], 50.0% [19], 98.6% [17], and 99.6% [20] respectively. The PPV was reported in one of the cardiac arrest-studies as 26.8% (95% CI 25.9–27.7%) [17]. In these five studies the sensitivity and specificity relate to identification of cardiac arrest among patients that the ambulance personnel reported as cardiac arrest, not to a sample of unselected calls.

### Identification of acute coronary syndrome (ACS)

Among patients calling with chest pain, Gellerstedt et al. demonstrated a sensitivity of 82.6 and 17.4% false negatives when identifying acute coronary syndrome [21].

### Identification of stroke

The sensitivity for identifying stroke was 67.8% [22], 66.2% [23] and 77.7% [24], all three articles using local adaptations of the Cincinnati Prehospital Stroke Score, and 86.4% [25] using the MPDS Stroke Diagnostic Tool. While the specificity was 26.6% [25] for the Stroke Diagnostic Tool. Krebes et al. implemented a new algorithm based on the MPDS algorithm, and reported a sensitivity of 53.3% [26].

The PPV was 20.0% and the NPV 90.2% for the Stroke Diagnostic Tool [25], and 30.2% PPV for the adapted Cincinnati Prehospital Stroke Score [23]. The PPV was 47.8% with the new algorithm by Krebes [26].

Over-triage was 11.6% for the adapted Cincinnati Prehospital Stroke Score and 20.8% for the National Guidelines for Telephone Triage Tool and under-triage 10.7 and 13.6% respectively [24].

In these five articles the sensitivity and specificity relate to identification of stroke among patients with the hospital diagnosis of stroke, not to a general population of unselected calls.

### Identification of major trauma

Only publications addressing the use of HEMS in the context of major trauma met with the inclusion criteria, which is why other publications addressing major trauma were not included in the current review. The dispatch criteria for HEMS had a sensitivity 87.7%, a specificity of 45.3%, a PPV of 48.4%, and a NPV of 86.3% for the HEMS dispatch criteria to identify major trauma patients [15]. Wilmer et al. described the different dispatching methods within the same dispatching system to study the accuracy of the systems for dispatching HEMS for major trauma [27]. Mechanism of injury together with the paramedic interrogation had a sensitivity of 80.2% and under-triage of 19.7%.

Two studies [15, 21] are in part derivation studies; i.e. studies with the aim of deriving a more accurate dispatching system. The data included in the current review from these articles is that reflecting the dispatching system in use, not the derived and unevaluated new dispatching system.

### Identification according to medical priority

The overall sensitivity of identifying time critical conditions defined as ambulance dispatch priority 1 was 93.32% [28], for dispatching priority 1 and 2 in accordance with the standard of Medical Emergency Triage and Treatment System-A, METTS-A, red, orange and yellow, 95.9% [29]. In two studies, using Advisory Committee for Aeronautics (NACA) score, the overall sensitivity was 87% [14], and 86% [12] respectively. The sensitivity was 78.0% [30] using local criteria. While the specificity was 48% [13], 48.67% [29], 67% [14], 83.8%, and [30]. Ek et al. showed a specificity of 15.4% for priority 3 dispatching in accordance with METTS-A green and blue [29].

The reported predicted values were; PPV of 36.6% (CI 35.8–37.5%) and NPV of 96.9% (95% CI 96.8–97.1%) [30]. In Dami et al. PPV was 21.7% (21.2–22.2%) and NPV was 95.4 (95.2–95.6%) [12]. Ball et al. reported PPV of 5.85% (CI 5.71–5.99%) and NPV of 0.47% (95% CI 0.43–0.51%) [28]. Over-triage rate was 78% [13] and 71% [14] and under-triage rate was 4.6% [13] and 3% [14] respectively.

## Discussion

The results of the current study show that there is a very low to low overall level of evidence for the accuracy of medical dispatching systems. Although all the articles included in the current systematic review are primary research, it was not possible to perform a meta-analysis due to the heterogeneity of the sample. Moreover, it is striking that only two of the 18 articles included in the current review presents information on all measures of dispatching accuracy together with over-and under-triage, and there was only one prospective study [24]. We suggest that it may be necessary to create a consensus on common standards for reporting before consensus can be formed for the level of accuracy in medical dispatching systems.

### Identification of cardiac arrest

Identification of cardiac arrest is based on the recognition that the patient is unconscious and has abnormal or no breathing. Two studies in the review reported higher sensitivity following the implementation of new protocols [19, 20]. Interestingly, both these new protocols included keywords that reflect cardiac arrest in the call between the caller and the telecommunicator. Other more novel approaches are to focus on the communication in the emergency call [31]. The observation that it is important to evaluate the communication even when measuring accuracy, was demonstrated in the study by Möller et al., the sensitivity of identifying cardiac arrest was increased by listening to the actual calls [18].

### Identification of stroke

Identification of stroke has several challenges and as a result about half of the patients with stroke are identified by the medical dispatcher [32, 33]. Firstly the symptoms are often non-specific, as demonstrated by Clawson et al., in that more than one in ten patients have the chief complaint “sick person” and an additional one in ten have fallen [25]. Secondly, the objective for medical dispatching is not absolute. What is more important? Whether it is to identify an acute stroke [23–26], to identify that a patient needs to be directed to a stroke center or to identify the patient that is benefited by specific treatment, e.g. thrombolysis [22] or thrombectomy, remains undetermined. It is not possible to suggest a dispatching system which is superior based on the results of the current review since the level of evidence is very low and the outcome measures are different for the five included articles focusing on stroke identification.

### Identification of major trauma

Only publications addressing the use of HEMS in the context of major trauma met with the inclusion criteria, which is why other publications addressing major trauma were not included in the current review. HEMS is part of the chain of care for major trauma in resource strong settings. There is evidence that HEMS is of value for multitrauma patients and patients with traumatic brain injury [34–37] and is dispatched when medical intervention is thought to be needed [15]. However, over-triage is a problem. Up to every other deployment is cancelled, predominantly by ground EMS [15]. The accuracy of the medical dispatching could be increased by including vital signs and anatomical location of injury to the mechanism of injury which is the basis for the routine HEMS dispatching system [15]. While Wilmer et al. could show that the accuracy of HEMS dispatching was superior and comparable for paramedical interrogation of caller and the assessment of need by land ambulance crew as compared to the dispatching by mechanism of injury [38]. The results of these studies lead us to believe that mechanism of injury are insufficient criteria for HEMS dispatching for major trauma, although these results need to be interpreted with caution since the level of evidence is (very) low.

### Identification according to medical priority

Patients present to the telecommunicator with a wide range of symptoms, and the ultimate question is how to identify what resources are best needed for the given caller/ patient. Ball et al. considered the effect of the chief complaint in relation to over-and under-triage. The results showed that while some of the most common chief complaints are under-triaged, e.g. convulsions/ seizures and breathing problems, others are over-triaged e.g. chest pain, heart problems/ automatic defibrillator, collapse and headache. While systems with a large proportion of non-specific presentations will not be able to evaluate the system in detail [13]. Although more than half of the calls are dispatched as priority 1 - only approximately 5% of these calls are critical [27], demonstrating the large over-triage in systems, and at the same time, revealing the lack of consensus on what level over-triage level is reasonable.

There is scant evidence concerning the necessary skills and competence for the telecommunicator. An exception is the study by Leopardi et al., demonstrating that experienced nurses could assess the patients’ need for advanced care as well as a medical doctor [30]. The required level of competence of telcommunicators is an area in need of further research.

### Over-and under-triage and the accuracy of medical dispatching systems

We gear our emergency response systems so as not to miss patients in need of medical intervention -i.e. to avoid under-triage - and compensate by creating over-triage, i.e. “unnecessary” dispatching. Dispatching systems are e.g. “front loaded”, i.e. over-triage is used as a safety rule and we assume that by creating over-triage we are “safe”. However, that this is not the case is illustrated by HEMS having an over-triage of 44% and simultaneously, in the same dispatching system, an under-triage of 20% [15]. It is therefore clear that although we need to understand and set cut-off levels for over-triage (so as to avoid waste of resources and risk for personnel) and under-triage (so as to avoid potentially lifesaving interventions not being given), they are - as measures of a dispatching system – insufficient on their own.

Measures of accuracy for dispatching systems are needed as a step in the direction of getting the right treatment to the right patient at the right time. However, there is an inherent challenge to identify the subset of patients that benefit from a specific intervention e.g. HEMS or acute coronary syndrome [21, 31]. In addition to making sure that e.g. the patient with a stroke can arrive in a timely fashion to the stroke center, this will also allow for telephonic support for interventions e.g. stopping a major bleeding or to perform CPR. Such studies are designed with the aim of including parameters that increase the accuracy of identification of specific conditions or diagnoses.

In addition to identifying specific conditions, it is also important to identify time critical conditions among patients presenting with a broad range of symptom presentations and to dispatch according to medical priority, i.e. without a definite diagnosis. There are no obvious answers to the best way forward. However, to agree on how to measure and report on dispatching systems is necessary in order to be able to compare different systems between different populations and settings. There are suggestions [38, 39], but these consensus documents have not been applied in the current literature, and it is time to take this a step further.

### Limitations

In 2011 Fevang et al. published a consensus report on the top five research priorities in pre-hospital care [40]. Among suggested topics was dispatch system accuracy. The choice of this was based on dispatching accuracy being a well-defined aim, with defined outcome measures, pertinent operational ramifications, and an area where there was a sufficient number of published articles which made the systematic review possible. It is possible that the search was additionally limited by using specific search terms, however, the search was broad as presented in Table 1.

The definitions of the measures of accuracy and over-and under-triage are not the same in the included articles, which limits comparisons of the results from the different articles. Although sensitivity was defined as the probability of the medical dispatching system identifying a specific condition given that this condition is present; specificity and the predictive values did not have the same definition. Specificity was often defined in relation to a specific condition, and not in relation to an unselected sample of callers without this specific condition. Also, the definition of over- and under-triage differed between the articles. That the definition of the measures varies makes comparisons of the results difficult.

Additional factors making comparisons between the different studies difficult are e.g. that there are two in principal different categories of dispatching systems/protocols; i.e. the MPDS and the CBDS. Moreover, the responding EMS has different tiers and organizations, again; leading to a lack with respect to a golden standard for outcome measures.

The level of evidence was categorized in accordance with GRADE, and in accordance with GRADE, retrospective studies are in general very low level of evidence. Although the overall level of evidence in the articles included in the current review was very low to low; the studies are informative and often necessary in order to design future studies. Following standards e.g. those set by STARD [41] should increase the quality of evidence.

It is imperative that the data collected from the electronic health care records is both valid and reliable before we can use this data in the design of clinical decision systems for medical dispatching. None of the reviewed studies analyzed the quality of data from the health records.

## Conclusions

There were 18 articles addressing the identification of cardiac arrest, stroke, medical priority and major trauma using different dispatching systems. The results of the current study show that there is an overall very low to low level of evidence for the accuracy of medical dispatching systems. We suggest that it is necessary to create a consensus on common standards for reporting before consensus can be reached for the level of accuracy in medical dispatching systems.

## Abbreviations

CBD: Criteria-Based Dispatch
EMS: Emergency medical services
GRADE: Grading of Recommendations Assessment, Development and Evaluation
HEMS: Helicopter emergency services
MeSH: Medical Subject Heading
METTS-A: Emergency Triage and Treatment System-A
MPDS: Medical Priority Dispatch system
NACA: Advisory Committee for Aeronautics
NPV: Negative predictive value
PPV: Positive predictive value
PRISMA: Systematic Reviews and Meta-Analyses
PROSPERO: International prospective register of systematic reviews
